# Supplementary figures and images for: AI-Enhanced Fluorescein Angiography Detection of Diabetes-Induced Silent Retinal Capillary Dropout and RNA-Seq Identification of Pre-Symptomatic Biomarkers
Source: Biomedicines. 2025 Aug 7;13(8):1926. doi: 10.3390/biomedicines13081926 (PMC12383729; doi:10.3390/biomedicines13081926)

Supplementary Figure S1

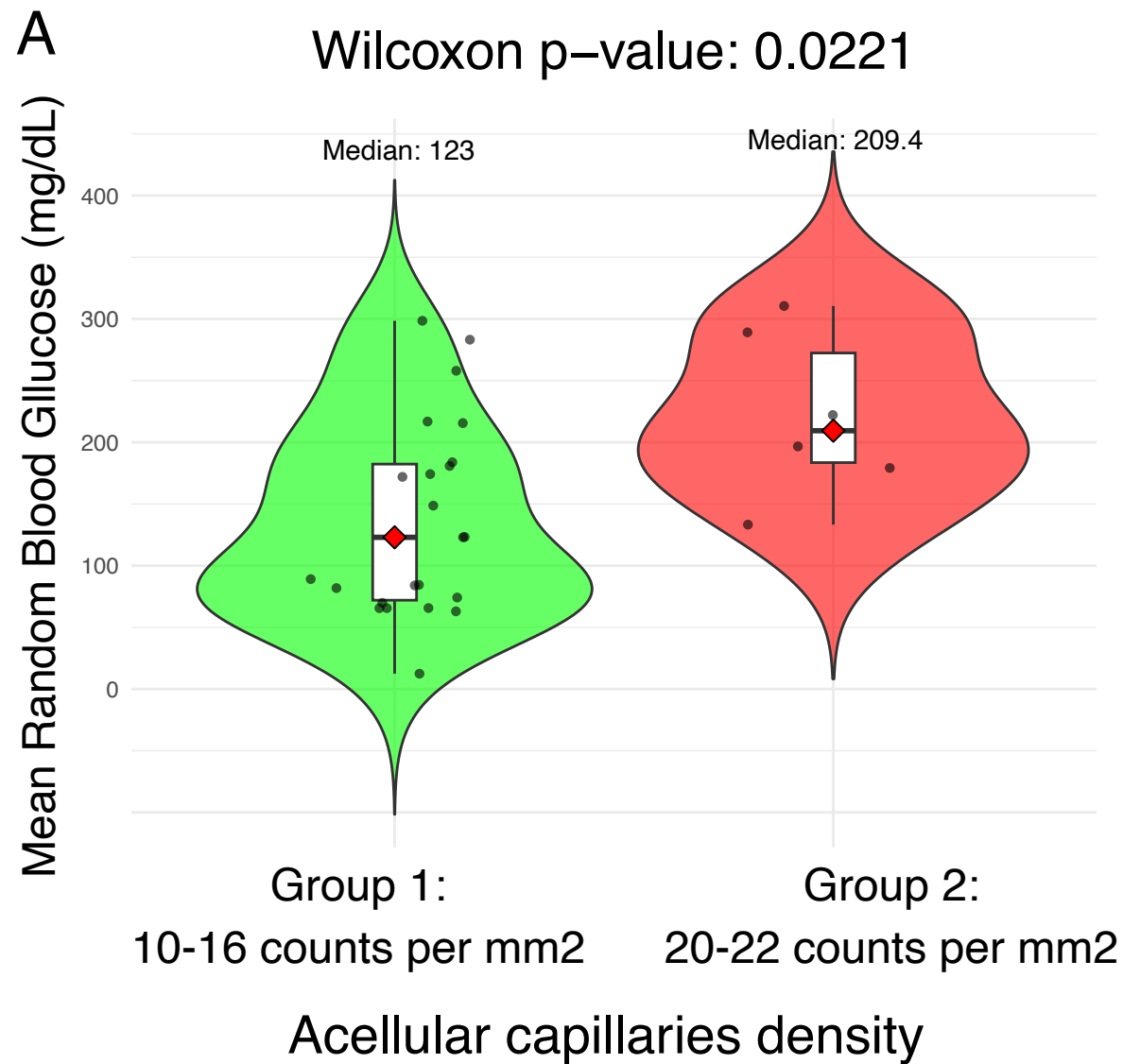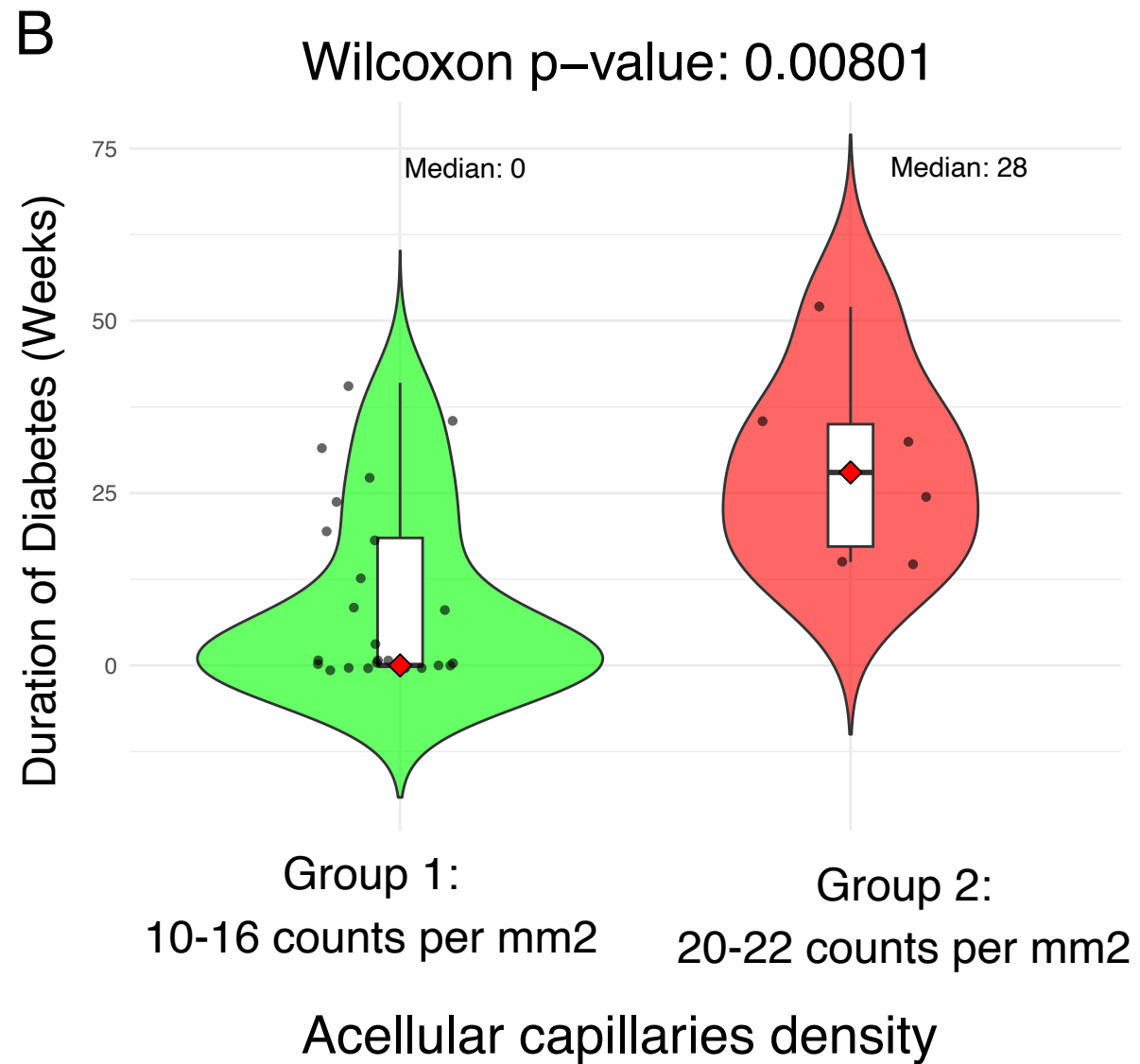

Supplement: Supplementary file 1 [file biomedicines-13-01926-s001.zip › biomedicines-3753105-supplementary.pdf]
